# Diagnostic Value of Erythrocyte Sedimentation Rate and C Reactive Protein in detecting Diabetic Foot Osteomyelitis; a Cross-sectional Study

**Published:** 2020-09-08

**Authors:** Seyed Kaveh Moallemi, Mahtab Niroomand, Niki Tadayon, Mohammad Mehdi Forouzanfar, Alireza Fatemi

**Affiliations:** 1 AJA University of Medical Sciences, Tehran, Iran.; 2 Division of Endocrinology, Department of Internal Medicine, Shahid Beheshti University of Medical Sciences, Tehran, Iran.; 3 Surgery Department, Shahid Beheshti University of Medical Sciences, Tehran, Iran.; 4 Emergency Department, Shohadaye Tajrish Hospital, Shahid Beheshti University of Medical Sciences, Tehran, Iran.; 5 Men's Health and Reproductive Health Research Center, Shahid Beheshti University of Medical Sciences, Tehran, Iran.

**Keywords:** C-reactive protein, Diabetic foot, Blood Sedimentation, Osteomyelitis

## Abstract

**Introduction:**

Osteomyelitis is one of the complications of diabetic foot infection. The present study aimed to evaluate the diagnostic value of erythrocyte sedimentation rate (ESR) and C reactive protein (CRP) in detection of osteomyelitis in patients with diabetic foot.

**Methods:**

In this cross-sectional study, serum levels of ESR and CRP were measured for patients with diabetic foot referring to emergency department or endocrinology clinic and the screening performance characteristics of these markers in detection of osteomyelitis were calculated. The diagnosis of osteomyelitis was based on clinical examination and positive probe-to-bone test, which was confirmed by plain x-rays or MRI.

**Results:**

142 diabetic patients with an average age of 61.2 ± 11.8 years were evaluated (66.2 % male). The area under the ROC curve of ESR in detection of osteomyelitis in diabetic foot cases was 0.70 (95% CI: 0.62-0.79). The best ESR cut-off point in this regard was 49 mm/hour. Sensitivity, specificity, positive and negative predictive values, and positive and negative likelihood ratios of ESR in 49 mm/Hour cut-point were 74.6% (95% CI: 62.9-83.9), 57.7% (95% CI: 45.5-69.2), 63.9% (95% CI: 52.5-73.9), 69.5 % (95% CI: 56.0-80.0), 1.8 (95% CI: 1.3-2.4) and 0.4 (95% CI: 0.3-0.7), respectively. The area under the ROC curve of CRP in detection of osteomyelitis was 0.67 (95% CI: 0.58-0.76). The best cut-off point for CRP in this regard was 35 mg/liter with sensitivity, specificity, positive and negative predictive values, and positive and negative likelihood ratios of 76% (95% CI: 64.2-85), 54.9% (95% CI: 42.7-66.6), 62.8% (95% CI: 51.6-72.8), 69.6% (95% CI: 51.7-80.8), 1.7 (95% CI, 1.3-2.2), and 0.4 (95% CI: 0.3-0.7), respectively.

**Conclusion:**

Based on the findings of ROC curve analysis, ESR and CRP had fair and poor accuracy, respectively, in detecting the diabetic foot cases with osteomyelitis.

## Introduction

More than 50% of non-traumatic amputations in the United States are lower-extremity amputations due to diabetes mellitus (1). About 5% of diabetic persons are facing foot ulcers each year and treatment of foot ulcers accounts for 15-25% of total health care processes for diabetes (2, 3). Chronic and deep wounds can get complicated by osteomyelitis as a consequence of contiguous spread of the microorganisms from soft tissues to periosteum, and that is the main cause of amputation in the majority of these patients (4). 

Timely diagnosis of osteomyelitis is important for preventing migration of the infection, and delay in osteomyelitis wound treatment leads to recurrent foot ulcers, increases the likelihood of need for surgical intervention and amputation, and elongates the duration of antibiotic therapy (5, 6). Several different options, including computed tomography (CT) scan, magnetic resonance imaging (MRI), and triple-phase bone scans, with different degrees of sensitivity and specificity, have been introduced for use in diagnosis of osteomyelitis (7), but some studies have revealed that such imaging modalities were not cost-effective and, therefore, suggested that clinical signs of infection coupled with the findings of the wound probing to bone can be much more cost-effective for this purpose (8).

Increased serum inflammatory markers such as White blood cells (WBCs), C-reactive protein (CRP), and erythrocyte sedimentation rate (ESR) have been proposed to aid in diagnosis of osteomyelitis. However, in patients with diabetes, their value in the presence of infection has been questioned (9, 10). The aim of this study was to determine the diagnostic accuracy of ESR and CRP in detection of diabetic foot cases with osteomyelitis. 

## Methods:

***Study design and setting***

In this cross-sectional study, after receiving the ethics approval (ethics code: IR.SBMU.RETECH.REC.1396.540) and patient informed consent, all patients who met the American Diabetes Association Criteria (11) for diagnosis of diabetes mellitus and had diabetic foot, and had presented to the emergency department or endocrinology clinic of Shohadaye Tajrish Hospital, Tehran, Iran, from April 2018 to 2019 were evaluated.

***Participants***

Using convenience sampling, all >18 years old patients with diabetic foot were enrolled. Patients were excluded if they had any other concurrent infections, non-infectious inflammatory disease, or rheumatologic diseases. Patients who received immunosuppressive therapy and pregnant cases were also excluded.

***Data gathering***

Using a predefined checklist, age, gender, leukocyte count, serum level of ESR or CRP, the characteristics of the wound (bone visibility, positive probe test, dactylitis, wounds remaining more than four weeks, or septic secretions for more than two weeks), as well as presence or absence of osteomyelitis were registered for all cases. The diagnosis of osteomyelitis was based on clinical examination and positive probe-to-bone test and it was confirmed by plain X-rays or MRI (6). 

Six milliliter of peripheral blood sample was drawn from each individual and inserted into a vial containing Ethylenediamine Tetraacetic ethylenediaminetetraacetic acid (EDTA) anticoagulant. Blood samples were collected under aseptic conditions from an antecubital vein for determination of white blood cell (WBC) count and ESR using the modified Westergren method. Serum CRP was measured using BN ProSpec System (Siemens Healthcare Diagnostic Products GmbH, Marburg, Germany; inter- and intra-assay coefficient of variation <4%). 

***Statistical analysis***

Data were analyzed using Statistical Package for the Social Sciences (SPSS) version 16 (SPSS Inc. Chicago, IL) for Windows. Data were presented as mean ± standard deviation or frequency and percentage. The area under the receiver operating characteristic (ROC) curve was used to determine the best cut-off point of studied markers in detecting osteomyelitis. P value of less than 0.05 was considered statistically significant. Accuracy of 90% - 100% was considered as excellent, 80% – 90% as good, 70% – 80% as fair, 60% – 70% as poor and < 60% as fail.

## Results

***Baseline characteristics of studied cases***

A total of 142 diabetic patients with an average age of 61.2 ± 11.8 years were evaluated (66.2 % male). The diagnosis of osteomyelitis was confirmed for 71 (50.0%) cases. The mean age of patients with and without osteomyelitis was 59.7 ± 11.5 and 62.7 ± 11.9 years, respectively (p = 0.12). Table 1 compares the baseline characteristics of patients with and without osteomyelitis. The frequency of open wound during the previous 4 weeks (64.8%; p = 0.03), presence of discharge during the last 2 weeks (47.9%; p <0.0001), and the prevalence of smoking (28.2%; p = 0.02) were significantly higher in osteomyelitis cases.

Serum ESR levels were 71.2 ± 29.8 and 50.8 ± 24.5 (mm/ hour) in cases with and without osteomyelitis, respectively (p < 0.0001). These measures were 65.8 ± 37.2 and 43.6 ± 33.0 (mg/liter), respectively, for CRP level (p = 0.0002). The number of white blood cells was significantly higher in the osteomyelitis group (p = 0.005). 

***The diagnostic values of ESR and CRP***

**Figure1:**
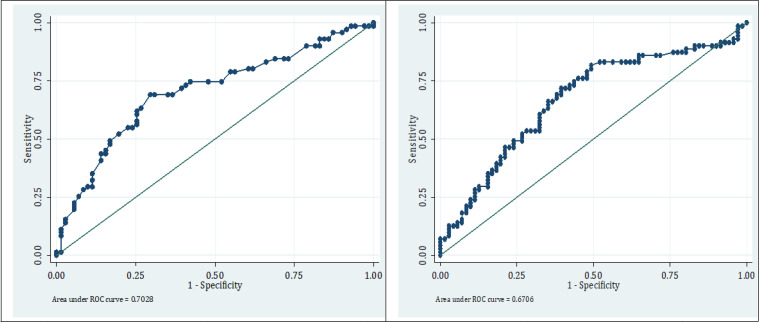
The area under the receiver operating characteristic (ROC) curve of erythrocyte sedimentation rate (left) and C reactive protein (right) in detecting the diabetic foot cases with osteomyelitis

Area under the ROC curve of ESR in detection of osteomyelitis in diabetic foot cases was 0.70 (95% CI: 0.62-0.79; figure 1). The best ESR cut-off point in this regard was 49 mm/hour. The sensitivity, specificity, positive and negative predictive values, and positive and negative likelihood ratios of ESR in 49 mm/Hour cut-point were 74.6% (95% CI: 62.9-83.9), 57.7% (95% CI: 45.5-69.2), 63.9% (95% CI: 52.5-73.9), 69.5 % (95% CI: 56.0-80.0), 1.8 (95% CI: 1.3-2.4) and 0.4 (95% CI: 0.3-0.7), respectively. 

Area under the ROC curve of CRP in detection of osteomyelitis was 0.67 (95% CI: 0.58-0.76; figure 1). The best cut-off point for CRP in this regard was 35 mg/liter with sensitivity, specificity, positive and negative predictive values, and positive and negative likelihood ratios of 76% (95% CI: 64.2-85), 54.9% (95% CI: 42.7-66.6), 62.8% (95% CI: 51.6-72.8), 69.6% (95% CI: 51.7-80.8), 1.7 (95% CI, 1.3-2.2), and 0.4 (95% CI: 0.3-0.7), respectively.

**Table 1:** Comparing the characteristics of studied patients between cases with and without osteomyelitis

**Variables**	**Suffering from osteomyelitis**		**P **
	**Without n = 71**	**With n = 71**	
Smoking	9(12.7%)	20(28.2%)	0.02
Open wound in last 4 weeks	33(46.5%)	46(64.8%)	0.03
Discharge during last 2 weeks	7(9.9%)	34(47.9%)	< 0.0001
Visible bone	0(0%)	2(2.8%)	0.49
Atherosclerosis	35(49.3%)	31(43.7%)	0.50
Hypertension	36(50.7%)	40(56.3%)	0.50
Hyperlipidemia	15(21.1%)	20(28.2%)	0.33

Data are presented based on frequency (%).

## Discussion

Based on the findings of ROC curve analysis the ESR and CRP had fair and poor accuracy, respectively, in detecting diabetic foot cases with osteomyelitis. 

An infected bone in patients with diabetic foot ulcers causes major clinical complication and increases the risk of surgical procedures and lower extremity amputations and also increases the probability of treatment outcomes like antibiotic resistance, kidney injury and catheter-related adverse events, which limit options for treatment and therapy and worsen the prognosis for cure (12-14). In spite of these potentially devastating consequences, most treatment options are based on experience and advice, due to lack of high-quality evidence. The findings of this study showed that the predictive value of ESR and CRP in detecting osteomyelitis is desirable. The sensitivity and specificity for ESR and CRP were 74.6%, 57.7% and 76%, 54.9%, respectively. The value of each of these biomarkers is significantly less than MRI. 

Kaleta et al. (15) found that erythrocyte sedimentation rate was significantly higher in the presence of underlying bone infections. In addition, they determined the optimal cut-off value with the highest positive and negative predictive values as 70 mm/h. These finding confirm the role of ESR in prediction of osteomyelitis in diabetic patients; however, the best ESR cut-off point to predict osteomyelitis, was 49 mm per hour in the current study. 

Most previously published studies have failed to assess the relationship between ESR and presence of osteomyelitis or have not specifically studied its use in the diabetic population, while many of them have stated that elevation in ESR is a predictor of the presence of osteomyelitis (16-18). 

The results of Asten et al. study suggested a predictive role for both ESR and CRP at the time of monitoring the success of therapy in diabetic foot osteomyelitis (12).

The sensitivity and specificity of CRP (cut-off value >14 mg/L) in diagnosis of osteomyelitis were 0.85 and 0.83. These values were 0.84 and 0.75 for ESR (cut-off value > 67 mm/h), 0.75 and 0.79 for WBC (cut-off value >14 × 10^9^/L), and 0.81 and 0.71 for PCT (cut-off value >0.30 ng/mL), respectively, in Michail et al. study (2). Although there are some differences in values, but that study, like the present study, showed that serum inflammatory markers including CRP, ESR and WBC could be used for the diagnosis of foot infections in patients with diabetes.

Based on the Oktay Yapıcı et al. study (19), neutrophil-to-lymphocyte ratio, CRP, and ESR have a predictive value for development of osteomyelitis and progression to amputation in patients with diabetic foot osteomyelitis. 

As can be seen, all of the mentioned studies have reported a significant relationship between inflammatory markers and osteomyelitis. However, the reported diagnostic values for these markers vary between studies, with sensitivity ranging from 75% to 90% and specificity ranging from 70% to 80%. 

The findings of this study showed that on average, the values of all inflammatory markers, including ESR and CRP, show an increase in patients with diabetic foot infections and higher values were found in patients with osteomyelitis. 

## Limitations

Limitations of this investigation include the small number of cases reviewed. In future investigations, a prospective evaluation, in which all patients with osteomyelitis are confirmed with pathology, would improve the value of the study. In addition, the authors did not correlate location of the bone involvement with laboratory values. It may prove that the presence of infection in highly vascular areas of the foot or body may affect the sedimentation rate.

## Conclusion:

Based on the findings of ROC curve analysis, ESR and CRP had fair and poor accuracy, respectively, in detecting the diabetic foot cases with osteomyelitis. 
